# Improved precision in SASHA T_1 _mapping with a variable flip angle readout

**DOI:** 10.1186/1532-429X-16-S1-M9

**Published:** 2014-01-16

**Authors:** Kelvin Chow, Bruce S Spottiswoode, Joseph J Pagano, Richard B Thompson

**Affiliations:** 1 Department of Biomedical Engineering, University of Alberta, Edmonton, Alberta, Canada; 2 Cardiovascular MR R&D, Siemens Healthcare USA, Inc., Chicago, Illinois, USA

## Background

The SAturation-recovery single-SHot Acquisition (SASHA) T_1 _mapping sequence has excellent accuracy independent of T_1_, T_2_, heart rate, and flip angle [1], which are known dependencies of the more commonly used MOdified Look-Locker Inversion-recovery (MOLLI) sequence. However, SASHA has a greater T_1 _variability (poorer precision) compared to MOLLI. A two-parameter fit, with assumed ideal saturation, has been shown to improve precision compared to the standard three-parameter fit used for SASHA, but at the expense of introducing systematic errors [2]. We propose that a variable flip angle (VFA) readout will reduce these systematic errors and thereby allow the improved precision of a two-parameter fit while maintaining the accuracy of the three-parameter fit.

## Methods

A VFA scheme was empirically designed with Bloch equation simulations to minimize two-parameter fit errors with SASHA data, consisting of scaling the prescribed flip angle for the first 45 pulses by sin(x) for π/90 < × < π/2. The first 5 data acquisitions in the pulse train were discarded, matching the number of dummy pulses with linear catalyzation in the standard SASHA sequence. SASHA, SASHA-VFA, and MOLLI T_1 _imaging was performed on 4 healthy volunteers (Siemens Aera 1.5T) on a mid-ventricular short-axis slice with typical bSSFP imaging readout parameters: 1.01/2.44 ms TE/TR, 8 mm slice thickness, 112 × 192 matrix size, 270 × 360 mm^2 ^field of view, rate 2 GRAPPA with 24 in-place ACS reference lines, 78% phase resolution, and 7/8 partial Fourier for a total imaging duration of ~175 ms. SASHA datasets were acquired with 9 images having equally spaced TIs from 165-780 ms following BIR-4 saturation, plus a non-saturated image. Standard SASHA was acquired with 5 (dummy) linear catalyzation pulses and SASHA-VFA was acquired with sinusoidal scaling described above, both with a target flip angle of 70°. MOLLI data was acquired with a 5-(3)-3 configuration, 120 ms TI start, 80 ms TI increment, 35° flip angle, and a tan/tanh adiabatic inversion pulse [3]. T_1 _pixel map were generated and the mean and standard deviation calculated for an ROI enclosing the entire LV myocardium.

## Results

**Figure 1:**
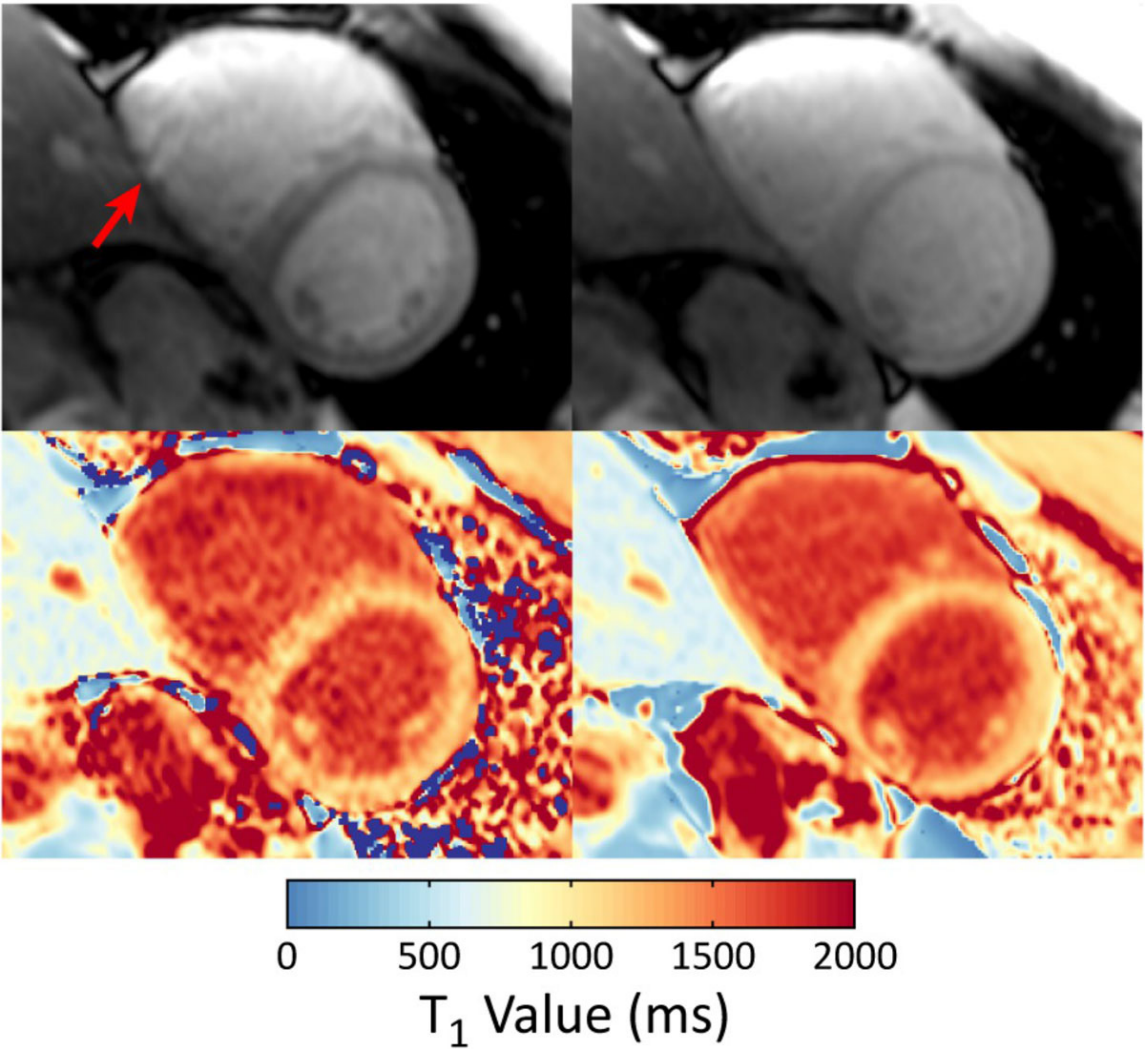
**Non-saturated images (top) and T_1 _pixel maps (bottom) for standard SASHA (left) and SASHA-VFA (right) in a healthy subject**. An artifact (arrow) in the inferior right ventricular wall is seen in the non-saturated image for standard SASHA, but not for SASHA-VFA.

Two-parameter SASHA overestimated myocardial T_1 _as compared to the three-parameter fit but with reduced variability (Table 1). Two-parameter SASHA-VFA showed similar mean T_1 _values to three-parameter SASHA and with substantially reduced T_1 _variability. Image artifacts from the bSSFP readout were consistently reduced with the SASHA-VFA sequence compared to the standard SASHA sequence, which may also contribute to the improved variability performance (Figure 1).

**Table 1:** Mean, standard deviation, and coefficient of variation of myocardial T_1 _values in 4 healthy subjects

	Mean Myocardial T_1 _(ms)	Standard Deviation of Myocardial T_1 _(ms)	Coefficient of Variation of Myocardial T_1 _(%)
SASHA (3-parameter fit)	1165 ± 15	78 ± 12	6.9 ± 1.0
SASHA (2-parameter fit)	1177 ± 29	58 ± 5	4.9 ± 0.3
SASHA-VFA (2-parameter fit)	1163 ± 19	47 ± 5	4.1 ± 0.5
MOLLI	996 ± 12	43 ± 4	4.3 ± 0.3

Values are reported as mean ± standard deviation across subjects.

## Conclusions

The SASHA sequence with VFA readout significantly reduces T_1 _variability and reduces image artifacts. The current study suggests that two-parameter SASHA-VFA maintains the accuracy of standard three-parameter SASHA with significantly reduced T_1 _variability, similar to the MOLLI sequence.

## Funding

Canadian Institutes of Health Research, Women and Children's Health Research Institute, Alberta Innovates - Health Solutions.

## References

[B1] Chow K MRM 2013 doi:10.1002/mrm.24878

[B2] Kellman P ISMRM 2013 21 1394

[B3] Kellman P MRM 2013 doi:10.1002/mrm.24793

